# Lifelong Obesity in a Polygenic Mouse Model Prevents Age- and Diet-Induced Glucose Intolerance– Obesity Is No Road to Late-Onset Diabetes in Mice

**DOI:** 10.1371/journal.pone.0079788

**Published:** 2013-11-13

**Authors:** Ulla Renne, Martina Langhammer, Julia Brenmoehl, Christina Walz, Anja Zeissler, Armin Tuchscherer, Marion Piechotta, Rudolf J. Wiesner, Maximilian Bielohuby, Andreas Hoeflich

**Affiliations:** 1 Laboratory for Mouse Genetics, Leibniz Institute for Farm Animal Biology, Dummerstorf, Germany; 2 Livestock Genetics and Breeding Unit, Institute for Genetics & Biometry, Leibniz Institute for Farm Animal Biology, Dummerstorf, Germany; 3 Clinic for Cattle, University of Veterinary Medicine, Hannover, Germany; 4 Center for Physiology and Pathophysiology, Institute of Vegetative Physiology, University of Cologne, Cologne, Germany; 5 Center for Molecular Medicine Cologne, University of Cologne, Cologne, Germany; 6 Cologne Excellence Cluster on Cellular Stress Responses in Aging-associated Diseases, University of Cologne, Cologne, Germany; 7 Endocrine Research Unit, Medizinische Klinik und Poliklinik IV, Klinikum der Universität, Munich, Germany; 8 Cell Signaling Unit, Institute for Genome Biology, Leibniz Institute for Farm Animal Biology, Dummerstorf, Germany; Haassah Medical Center, Israel

## Abstract

**Aims/Hypothesis:**

Visceral obesity holds a central position in the concept of the metabolic syndrome characterized by glucose intolerance in humans. However, until now it is unclear if obesity by itself is responsible for the development of glucose intolerance.

**Methods:**

We have used a novel polygenic mouse model characterized by genetically fixed obesity (DU6) and addressed age- and high fat diet-dependent glucose tolerance.

**Results:**

Phenotype selection over 146 generations increased body weight by about 2.7-fold in male 12-week DU6 mice (P<0.0001) if compared to unselected controls (Fzt:DU). Absolute epididymal fat mass was particularly responsive to weight selection and increased by more than 5-fold (P<0.0001) in male DU6 mice. At an age of 6 weeks DU6 mice consumed about twice as much food if compared to unselected controls (P<0.001). Absolute food consumption was higher at all time points measured in DU6 mice than in Fzt:DU mice. Between 6 and 12 weeks of age, absolute food intake was reduced by 15% in DU6 mice (P<0.001) but not in Fzt:DU mice. In both mouse lines feeding of the high fat diet elevated body mass if compared to the control diet (P<0.05). In contrast to controls, DU6 mice did not display high fat diet-induced increases of epididymal and renal fat. Control mice progressively developed glucose intolerance with advancing age and even more in response to the high fat diet. In contrast, obese DU6 mice did neither develop a glucose intolerant phenotype with progressive age nor when challenged with a high fat diet.

**Conclusions/Interpretation:**

Our results from a polygenic mouse model demonstrate that genetically pre-determined and life-long obesity is no precondition of glucose intolerance later in life.

## Introduction

Within the concept of the metabolic syndrome visceral obesity is considered as an important risk factor in humans [1]. In this concept abdominal fat is a precondition of altered adipokine and fatty acid serum profiles, which decrease insulin sensitivity as an early feature of the syndrome. Obesity itself results from the disequilibrium of energy input and energy consumption. Imbalance of energy metabolism may be due to modern lifestyle characterized by relative hyperphagia, physical inactivity or may also be due to genetic predisposition.

In monogenic or polygenic forms of obesity at present between 9 and 58 genetic loci are discussed in a functional context [2], [3]. Since monogenic forms of obesity have been estimated to account for only 5–10% of all genetic forms it is clear that obesity in the vast majority of the population is related to more than one gene locus. For the analysis of polygenic obesity a number of mouse models had been established in the past [4]. Most importantly growth selected *M16* [5], *Kuo Kondo* [6], polygenic *New Zealand Obese* [7] or *Tsumura Suzuki Obese Diabetes* [8] mice were characterized by obesity, hyperphagia and hyperglycemia and thus without exception supported the positive link of obesity and metabolic lapse. Also high fat diets (HFD) were used to characterize the interrelationships between acquired obesity and energy metabolism. Experiments using HFD to induce obesity and diabetes [9], [10] provided a link between these two outcomes or revealed both obesity- and diabetes-resistant strains [11], [12]. With the identification of benign obesity [13], [14] it was recognized that some obese patients may be as insulin sensitive as lean patients. However still the majority of obese patients is characterized by glucose intolerance. In order to address the question to which extent obesity is functionally linked with glucose insensitivity we have used a novel mouse model established by 37 years of phenotype selection for high body mass. In the course of more than 140 rounds of selections it is clear that a high number of genetic events have been enriched contributing to the phenotype of high body mass. Thus our mouse model provides a robust genotype comparable to human populations to test the strength of a putative intricate interrelationship between obesity and glucose intolerance.

## Materials and Methods

### Animals

All procedures were performed in accordance to national and international guidelines and approved by our own institutional board (Animal Protection Board from the Leibniz-Institute for Farm Animal Biology) and by the national Animal Protection Board Mecklenburg-Vorpommern (file number: LALLF M-V/TSD/7221.3-1.2-037/06). We used non inbred mouse lines established by phenotype selection over 146 generations for high body mass (DU6) at an age of 42 d after birth [15], [16]. In addition, an unselected control mouse line, Fzt:DU, based on the identical outbred stock (generation 153) was used. The initial population was derived from an original crossbred of four outbred (NMRI orig., Han:NMRI, CFW, CF1) and four inbred (CBA/Bln, AB/Bln, C57BL/Bln, XVII/Bln) populations. The mice were maintained at a temperature of 22°C and at 45% humidity in a semi-barrier system and had free access to a standard diet and water ad libitum. After weaning and during the experiment, 20 male mice per group were singly kept either on a standard chow diet (Altromin C 1080 Obesity & Diabetes control diet ) or on a HFD (Obesity & Diabetes high fat diet). Composition of both diets (Altromin, Lage, Germany) is provided in Table 1. Body weights were recorded in 3-week intervals between 3 and 39 weeks of age. At selected intervals (5- to 6-week, 11- to 12-week, 17- to 18-week and 23- to 24-week) during the experiment food consumption was monitored in both mouse lines and both diet groups. For the experiment defined amounts of food were distributed in cages of isolated male mice. The consumed food was recorded in weekly intervals. Dispersed food pellets in the cages were collected and added to the amount of residual food before weight recordings.

**Table 1:** Compositions of Altromin control chow and high fat diet (HFD; C 1080) used in the present study.

	Chow	HFD
Fat [g/kg]	46.00	236.00
Energy content of fat fraction [%kcal]	11.50	46.00
Carbohydrate [g/kg]	592.00	428.00
Energy content of carbohydrate fraction [%kcal]	66.00	37.00
Crude Protein [g/kg]	200.00	200.00
Energy content of protein fraction [%kcal]	22.50	17.00
Crude fiber [g/kg]	46.00	47.00
Ash [g/kg]	38.00	38.00
Vitamin A [IU/kg]	20000.00	20000.00
Vitamin D3 [IU/kg]	2000.00	2200.00
Vitamin E [mg/kg]	120.00	120.00
Vitamin K3 [mg/kg]	25.00	25.00
Vitamin B1 [mg/kg]	22.00	22.00
Vitamin B2 [mg/kg]	22.00	22.00
Vitamin B6 [mg/kg]	22.00	22.00
Vitamin B12 [mg/kg]	38.00	38.00
Pantothenic acid [mg/kg]	66.00	66.00
Nicotinic acid [mg/kg]	99.00	99.00
Folic acid [mg/kg]	2.00	2.00
Biotin [mg/kg]	0.44	0.44
Choline [mg/kg]	1925.00	1925.00
Calcium [mg/kg]	5900.00	5700.00
Phosphorus [mg/kg]	4400.00	4300.00
Magnesium [mg/kg]	550.00	550.00
Sodium [mg/kg]	1200.00	1200.00
Potassium [mg/kg]	3600.00	3600.00
Chlorine [mg/kg]	1600.00	1600.00
Iron [mg/kg]	48.00	47.00
Manganese [mg/kg]	53.00	52.00
Zinc [mg/kg]	36.00	36.00
Copper [mg/kg]	6.00	7.00
Iodine [mg/kg]	0.25	0.20
Selenium [mg/kg]	0.20	0.20
Cobalt [mg/kg]	0.02	0.02

Ether extracts from soybean oil and lard were used for fat isolation. Corn starch, maltodextrin, dextrose and sucrose were used for the preparation of the carbohydrate fraction. Casein was the source for the protein fraction and cellulose was used for crude fiber production.

### Parameters of physical activity

Physical activity in the open field was measured in male mice at an age of 42 days after birth (N>18). In addition, maximal physical activity was assessed by use of a treadmill as described previously [17].

### Oral glucose tolerance test

Oral glucose tolerance tests (oGTT) were performed in 3-week intervals between 3 and 39 weeks of age. Starting the day before oGTT, mice were fasted between 4 pm and 8 am. Mice were given an oral dose of 1 g/kg glucose dissolved in tap water. Concentrations of blood glucose during oGTT were assessed before and 10, 30, 60 and 120 min after the oral glucose bolus. A single drop of blood from the tail tip was used for measurement of glucose concentrations using a commercial glucometer (Roche, Penzberg, Germany). After the last oGTT mice were re-fed for one day and sacrificed by decapitation. The carcass is defined as the body after removal of head, skin and inner organs. Isolated muscles and distinct fat pads were weighed in addition to the carcass and liver. Tissues and serum samples were stored at -70°C.

### Analysis of insulin and triglycerides

Insulin was assessed in serum samples from fed male DU6 mice and controls at an age of 7, 16 and 29 weeks by using a Mouse-Insulin ELISA (DRG Instruments GmbH, Marburg, Germany) according to the instructions of the manufacturer. The concentration was calculated against an insulin standard curve with the Magellan software (Dortmund, Germany) using the cubic spline modus. The detection limit for insulin was 0.2 µg/l and the intra-assay coefficient of variation was 3.5%. The concentrations of hepatic triglycerides were assessed in male DU6 mice (29 weeks of age; N = 10) and controls (Fzt:DU; N = 10) using the LT-SYS Triglyceride kit (Labor+Technik, Berlin, Germany).

### Data analysis and statistics

The statistical analyses were generated using SAS for Windows, Version 9.2 (SAS Institute Inc., 2009, Cary, NC, USA). Descriptive statistics and tests for normality were calculated with the UNIVARIATE procedure of Base SAS software [18]. Data that could be considered as approximately normal were analyzed by ANOVA using the MIXED procedure of SAS/STAT software [19]. The ANOVA model for blood glucose contained the fixed factors line (levels: Fzt:DU, DU6), diet (levels: chow, HFD), age (levels: weeks 3, 6, 9, 12, 15, 18, 21, 24, 27, 33, 39), the repeated factor time (levels: 0, 10, 30, 60, 120 min) and all corresponding interactions. Repeated measures on the same animal were taken into account by the repeated statement in proc mixed using a compound symmetry block diagonal structure of the residual covariance matrix. For tissue data we used a model with the fixed effects line (levels: Fzt:DU, DU6), diet (levels: chow, HFD) and the interaction line*diet. The variables hepatic triglycerides (39^th^ week), food uptake, physical activity and body mass at day 42, epididymal fat mass at day 49 of chow-fed animals were analyzed by one-way ANOVA with factor line (levels: Fzt:DU, DU6). The model for insulin included the fixed effects line (levels: Fzt:DU, DU6), age (levels: weeks 7, 16 and 29) and corresponding interactions.

In addition, least-squares means (LSM) and their standard errors (SE) were computed for each fixed effect in the models, and all pairwise differences of LS-means were tested by the Tukey-Kramer procedure. Effects and differences were considered significant if P < 0.05.

## Results

### Success of phenotype selection and physical parameters

**Figure 1:**
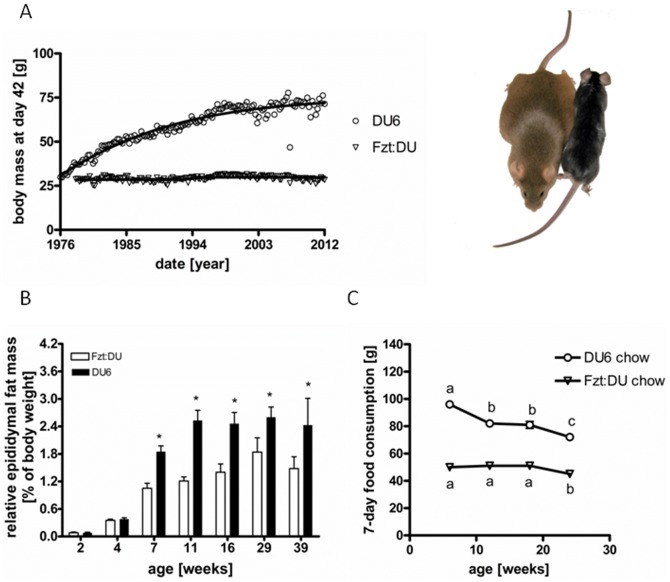
Establishment and basic features of the polygenic DU6 mouse model. Body weight selection was performed over 146 generations beginning in 1976 (Fig 1A). Epididymal fat mass (Fig. 1B) in male DU6 mice from 7 to 39 weeks of age (n = 15; *: P<0.01; in 39-week DU6 mice: n = 9). Longitudinal consumption of chow food (Fig 1C) was assessed in male mice in 4 different age groups over a period of 7 days (6, 12, 18 and 24 weeks of age; n>12; *: P<0.01).

**Figure 2:**
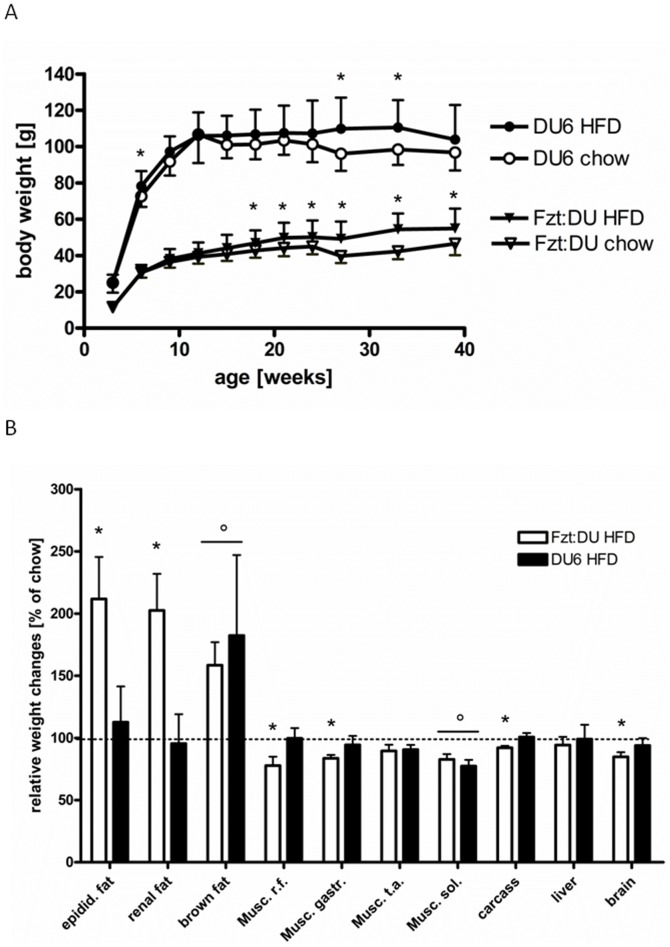
Changes in body and organ mass over life-time under chow and HFD. Effects of a HFD on body weight in male mice (Fig. 2A; until week 27: n>14; week 33 and 39: n>7; week 33: P<0.05). Fig 2B: Effects of a HFD on tissues masses in male mice at an age of 39 weeks (n>7; *: significant effect of line*diet P<0.01 vs. chow diet (100%); °: significant effect of diet as a main effect in both lines: P<0.05 (epidid. fat: epididymal fat mass; Musc. r.f.: Musculus rectus femoris, gastr: gastrocnemius, t.a.: tibialis anterior; sol.: soleus).

Phenotype selection for high body weight over a period of 37 years increased body weight in 7-week old male mice by 244% (Figure 1A). The overall phenotype of DU6 mice was characterized by a marked accumulation of epididymal fat (up to 5-fold increases in male DU6 mice; P<0.0001). Remarkably, the relative increases of epididymal fat mass exceeded the increases of total body mass at time points later than 4 weeks of age and were detected until 39 weeks of age (Fig. 1B; P<0.001). At all ages assessed, DU6 mice consumed more chow food if compared to age matched control mice (Fig. 1C; P<0.001). In DU6 mice but not in Fzt:DU mice food consumption was significantly reduced from week 6 to week 12 (P<0.001). If normalized for absolute body weight food consumption was reduced at all ages in DU6 mice (by up to 39% at an age of 12 weeks; P<0.001) as compared to age-matched Fzt:DU mice. Absolute consumption of HFD also was on a higher level in 6-week DU6 mice (83±15 g) if compared to 6-week Fzt:DU mice (33±10 g; P<0.001). Again, if compared to 6-week mice HFD intake was reduced in 24-week DU6 mice (55±7 g; P<0.001) but not in 24-week Fzt:DU mice (40±14 g; P<0.01). If compared to Fzt:DU mice the increases of body weight of DU6 mice ranged between 2.1-fold and 2.7-fold in 3-week and 12-week mice, respectively (Fig 2A). Feeding HFD was capable to further increase body weights in adult mice of both lines (P<0.05). In control mice but not in DU6 mice at an age of 39 weeks, HFD increased normalized masses of epididymal and renal fat (P<0.05; Fig 2B). As a main effect in both lines, HFD increased masses of brown fat (P<0.05) and reduced masses of M. soleus (P<0.05) if normalized to total body weight. Due to stronger increases of total body mass in Fzt:DU mice, normalized weights of isolated muscles, carcass and brain were significantly reduced (P<0.05). Mice did not have running wheels in their cages and thus cannot perform enhanced physical activity. DU6 mice, as observed empirically by the experienced technical staff that has developed the mouse model, have been described as being less active if compared to Fzt:DU mice. This assessment is in line with reduced open field activity of male DU6 mice (7.4±3.3 m/min) if compared to unselected controls (11.5±5.4 m/min) and reduced maximal running capabilities (DU6: 685±105 m versus Fzt:DU: 1865±963 m). However, both differences were statistically not significant.

### Longitudinal oral glucose tolerance tests (oGTT)

**Figure 3:**
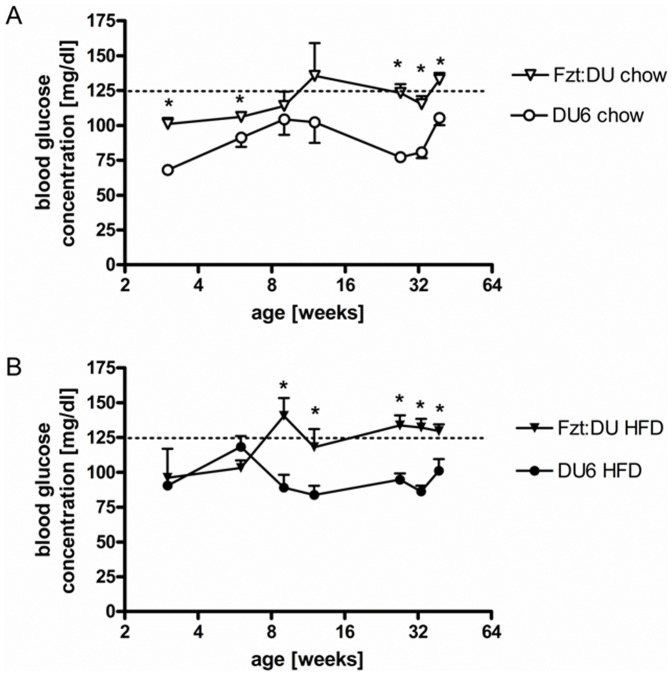
Longitudinal study of fasting glucose levels in male DU6 mice fed chow (A) or a high fat (B) diet (n_week3_ = 4_;_ n_week27_>13; *: P<0.05).

Both genetic groups were characterized by an age-related increase of fasting glucose levels under control diet (P<0.05; Fig 3). This increase was also found in control mice (P<0.05) fed a HFD but not in DU6 mice. With the exception of 6-week DU6 mice fed the HFD, DU6 mice showed reduced fasting glucose levels if compared to control mice (P<0.01).

**Figure 4:**
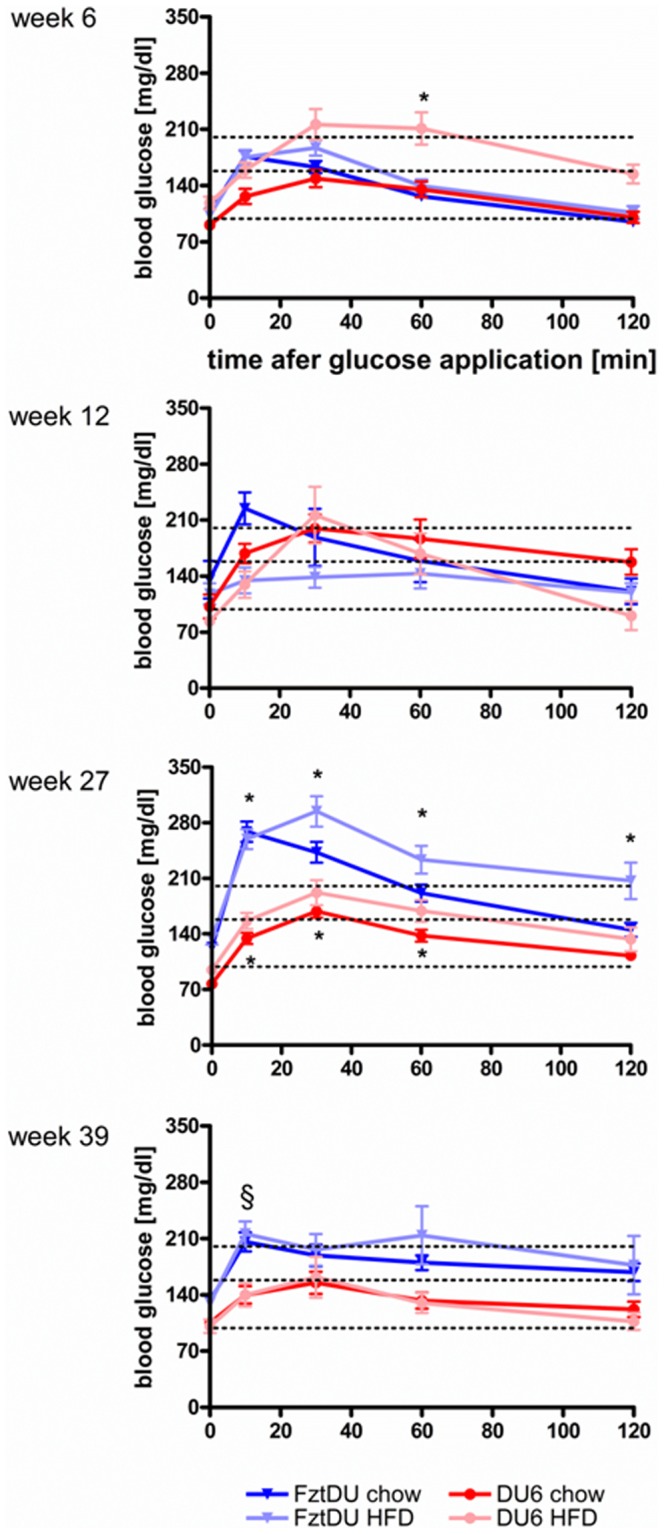
Longitudinal glucose tolerance tests in male DU6 and Fzt:DU mice under standard chow or HFD (n>14; *: significant interaction of line, diet, age and repeated measures of glucose analysis with P<0.05; §: significant interaction of line and age with P<0.001).

In DU6 mice, HFD impaired glucose tolerance maximally at an age of 6 week, with blood glucose concentrations exceeding the 200 mg/dl border 30 and 60 min after oGTT (Fig. 4, 1^st^ panel, pink line; P<0.05 if compared to control diet). Surprisingly, at later time points glucose tolerance progressively improved in DU6 mice. While no signs of age- or diet-dependent glucose intolerance were present in DU6 mice, control mice at an age of 27 weeks clearly had developed age- and diet-dependent glucose intolerance: under both diets Fzt:DU had excessively high glucose levels after oGTT (more than 250 mg/ml blood glucose), with retarded glucose clearing under the HFD (Fig. 4, 3^rd^ panel, chow: 145±37 mg/ml; HFD: 206±86 mg/ml 120 min after oGTT; P<0.05). If tested for the effect of mouse line within identical diet, age and time groups, significantly reduced concentrations of blood glucose were found in 27-week male DU6 after oGTT (P<0.05) at distinct time points under both diets if compared to control mice. Significant effects of line*age group interactions on blood glucose levels were present also in 39-week male DU6 mice (P<0.001) but not at an age of 6 or 12 weeks.

### Concentrations of serum insulin and triglycerides in the circulation and in the liver

In 7- and 16-week male DU6 mice serum insulin (7 weeks: 4.1±2.5 µg/l; 16 weeks: 3.6±2.0 µg/l) was increased (7 weeks: P<0.05; 16 weeks: P = 0.08) if compared to Fzt:DU mice (7 weeks: 1.5±0.9 µg/l; 16 weeks: 1.6±1.1 µg/l). At an age of 29 weeks in DU6 mice significant reductions of serum insulin concentration have been identified (DU6: 2.0±0.9 µg/l) if compared to 7-week male DU6 mice described above (P<0.05). In 29-week male Fzt:DU mice concentrations of insulin in serum were similar (Fzt:DU: 1.7±1.3 µg/l) if compared to 7-week Fzt:DU mice. At an age of 29 weeks DU6 mice had higher serum triglycerides if compared to random selected control mice (DU6: 1.8±0.5 µg/µl; Fzt:DU: 1.3±0.3 µg/µl; P<0.05). Hepatic triglycerides were similar in both genetic groups at the same age (DU6: 14.2±2.9 µg/mg; Fzt:DU: 12.9±3.8 µg/mg; n.s.).

## Discussion

### A novel mouse model to study the effects of elevated fat mass

We have developed a giant mouse model characterized by marked fat accretion (DU6). In some animals body weights of more than 135 g have been recorded, which to the best of our knowledge reaches the borders of mouse growth and cannot be exceeded by other growth selected mouse lines or the ectopic expression of growth hormone in transgenic mice [20]. At an age of 29 weeks DU6 mice are characterized by hyperlipidemia with no signs of hepatosteatosis. As published before also total cholesterol, HDL and LDL are elevated in obese DU6 mice [21]. Male DU6 mice are not characterized by increased physical activity but tended to display a less active phenotype if compared to unselected controls. The selection experiment is spanning 37 years now, corresponding to 146 repeated rounds of selections and has been described in detail before [22], [23]. It needs to be emphasized that the experiment was performed including about 70 crosses per generation and a mating scheme granting for the least effect on inbreeding. From the persistent success of selection we can assume that enrichment of a high number of alleles is contributing to the prominent phenotype of high body mass. In other words, due to a broader genetic background results obtained from DU6 mice are not restricted to a distinct e.g. inbred genotype.

### Food intake and obesity

In absolute terms DU6 mice consumed more food at all time points assessed if compared to control mice, which is in accordance to earlier studies in DU6 mice performed at generation 70 [24]. Relative food consumption was lower in DU6 mice if compared to controls. Thus one might interpret that relative energy intake is lower in DU6 versus Fzt:DU mice, particularly later in life. Increased food intake in the growth period may well be related to the fast accretion of body mass and body fat in comparison to unselected controls. Since young DU6 mice are characterized by hyperleptinemia and hyperinsulinemia, increased differentiation of fat cells and fat accumulation had been discussed previously for DU6 mice [25]. Although on a higher level at all time points in DU6 versus control mice, food ingestion and serum insulin concentrations were severely reduced in 12-week DU6 but not in randomly selected Fzt:DU mice. Thus, DU6 mice may represent an attractive model for the molecular study of appetite control especially at younger ages. With respect to the conditions of elevated and reduced food intake but particularly to the period of transition from higher to reduced food consumption an increasing amount of effectors has been addressed in separate studies [26]. Concerning the control of food intake and energy metabolism we may ask in future studies if phenotype selection has affected efficacy of nutrient utilization or acts by secondary effects e.g. through metabolic programming occurring in the fetal or early postnatal period [27]. So far, current obesity related therapeutic approaches do not target manipulation of nutrient utilization efficiency [28] although it is well accepted that the control of energy flux represents an important feature of metabolism [29], [30]. In DU6 mice control of metabolic efficacy particularly through protein acetylation as suggested by Wang et al. [31] appears as an attractive field for future research.

### Longitudinal analysis of glucose tolerance in obese and unselected control mice

By the comparison of a number of mouse models currently used to study the effects of obesity on metabolism it was demonstrated that the genetic background has a particular relevance for the specific effects on glucose tolerance [4]. Several genetic [6]–[8], [32]–[34] or diet-dependent [9], [35] mouse models for obesity were associated with the development of glucose intolerance. However, the HFD was also capable to establish improved glucose tolerance [9], [36] and these models were diabetes- and obesity-resistant. Thus, until now it was impossible to answer the question if abdominal obesity is a precondition or even a cause for the development of glucose intolerance later in life. In order to test the functional relationship between obesity in juvenile and adult mice and increased risk of an establishment of glucose intolerance later in life we investigated fasting glucose levels and glucose tolerance in response to oral glucose application in mice fed chow and a HFD. Surprisingly, elder obese DU6 mice had severely reduced fasting glucose levels if compared to random selected control mice. While unselected controls under HFD progressively with age acquired higher fasting glucose levels, DU6 mice did not. Lower fasting glucose concentrations are indicative of robust mechanisms that control glucose uptake and release by peripheral tissues, which might be due to higher leptin or insulin levels [25], IGF-I concentrations [37] or other factors in DU6 mice. Furthermore, increased insulin sensitivity triggered by higher leptin serum levels in DU6 mice may be considered at least as part of the mechanism. However, hyperinsulinemia in young DU6 mice is normalized with advanced age, thus insulin in DU6 cannot explain the smooth control of glucose levels at higher ages. Due to the inactive phenotype of DU6 mice we also can exclude the possibility that elevated physical activity protects against glucose intolerance in DU6 mice as shown e.g. in humans [38]. In search of potential mechanisms involved in the superior control of glucose levels of DU6 mice we cannot exclude a function of adipose tissues itself, which due to their high abundance may have an active role during improved glucose disposal. Very clearly, the multitude of potential mechanisms requires a detailed analysis, which particularly will address the prominent reductions of food consumption and insulin concentrations in DU6 mice between 6 and 12 weeks of age. In addition, a number of mouse models also characterized by benign obesity [39], [40] may serve as excellent starting points for the identification of distinct mechanisms for benign obesity in DU6 mice.

Within the hypothetical concept of the metabolic syndrome defined for humans, abdominal obesity has been discussed as a central event in the cascade of elevated fatty acids and adipokines in the circulation altering the metabolic profile and finally resulting in glucose intolerance [1]. Should our findings from mice be applied to the human condition this might imply that the role of body fat for glucose intolerance requires a refined definition. As we demonstrated in the present study performed in a polygenic mouse model, obesity alone cannot trigger glucose intolerance. In DU6 mice visceral obesity is definitely no direct road to glucose intolerance later in life. Thus, DU6 mice may represent a useful model to study conditions of benign obesity [13], according to which distinct hepatic features manage to interfere with the “pathogenesis of metabolic diseases” [14]. In spite of higher epididymal fat mass and hyperlipidemia, liver triglyceride content is normal in DU6 mice, which in fact shows, that the liver effectively manages the increased demands of energy metabolism in this model. Therefore, energy metabolism deserves a separate project in a context with metabolic control in age- and diet-dependent settings.

### Conclusion

From our data in a polygenic mouse model characterized by a broad genetic background we conclude that genetically determined obesity is not sufficient to favor the onset of glucose intolerance or diabetes in mice. Abdominal obesity in DU6 mice seems to be related to highly efficient energy management and control mechanisms of glucose uptake associated with the protection against age- and diet-dependent glucose intolerance. During the “micro-evolutionary” experiment that has developed the DU6 mouse model, a “formula” was obviously invented keeping glucose metabolism under superior control with progressing ages even in the presence of hyperphagia, severe obesity and dyslipidemia.
